# Polyethylene Composites with Segregated Carbon Nanotubes Network: Low Frequency Plasmons and High Electromagnetic Interference Shielding Efficiency

**DOI:** 10.3390/ma13051118

**Published:** 2020-03-03

**Authors:** Ludmila Vovchenko, Ludmila Matzui, Viktor Oliynyk, Yurii Milovanov, Yevgen Mamunya, Nadezhda Volynets, Artyom Plyushch, Polina Kuzhir

**Affiliations:** 1 Department of Physics, Taras Shevchenko National University of Kyiv, Volodymyrska str., 64/13, 01601 Kyiv, Ukraine; vovch@univ.kiev.ua (L.V.); matzui@univ.kiev.ua (L.M.); 2 Department of Radiophysics, Electronics, and Computer Systems, Taras Shevchenko National University of Kyiv, Volodymyrska str., 64/13, 01601 Kyiv, Ukraine; oliynyk@univ.kiev.ua; 3 Institute of High Technologies, Taras Shevchenko National University of Kyiv, Volodymyrska str., 64/13, 01601 Kyiv, Ukraine; juri_milovanov@yahoo.com; 4 Institute of Macromolecular Chemistry, National Academy of Sciences of Ukraine48 Kharkivske Chaussee, 02160 Kyiv, Ukraine; ymamunya@ukr.net; 5 Institute for Nuclear Problems of Belarusian State University, 11 Bobruiskaya Str., 220006 Minsk, Belarus; nadezhda.volynets@gmail.com (N.V.); artyom.plyushch@gmail.com (A.P.); 6 Faculty of Physics, Vilnius University, Sauletekio 9, LT-10222 Vilnius, Lithuania; 7 Institute of Photonics, University of Eastern Finland, Yliopistokatu 7, FI-80101 Joensuu, Finland

**Keywords:** carbon nanotube, polyethylene, segregated composite, complex impedance, electromagnetic shielding

## Abstract

Polyethylene (PE) based composites with segregated carbon nanotubes (CNTs) network was successfully prepared by hot compressing of a mechanical mixture of PE and CNT powders. Through comparison with a composite comprising randomly distributed carbon nanotubes of the same concentration, we prove that namely the segregated CNT network is responsible for the excellent electrical properties, i.e., 10^−1^ S/m at 0.5–1% and 10 S/m at 6–12% of CNT. The investigation of the complex impedance in the frequency range 1 kHz–2 MHz shows that the sign of real part of the dielectric permittivity $\varepsilon_{r}^{\prime }$ changes from positive to negative in electrically percolated composites indicating metal-like behavior of CNT segregated network. The obtained negative permittivity and AC conductivity behavior versus frequency for high CNT content (3–12%) are described by the Drude model. At the same time, in contrast to reflective metals, high electromagnetic shielding efficiency of fabricated PE composites in the frequency range 40–60 GHz, i.e., close to 100% at 1 mm thick sample, was due to absorption coursed by multiple reflection on every PE-CNT segregated network interface followed by electromagnetic radiation absorbed in each isolated PE granule surrounded by conductive CNT shells.

## 1. Introduction

The electrically conductive polymer composite materials (CMs) have received considerable attention due to their multi-functional applications in many engineering and electronic fields [1,2]. Carbon nanomaterials (carbon nanotubes and nanofibers, graphite nanoplatelets and graphene) due to their excellent properties such as lightweight, high corrosion resistance, high electrical and thermal conductivity [3,4], are now intensely used as fillers in polymer matrix for the development of novel materials with tunable electric/dielectric properties for many applications, including energy storage, piezoresistive sensing, and electromagnetic interference (EMI) shielding [5,6,7,8,9].

Theoretical predictions and experiments have confirmed that the formation of a continual conductive network is the key issue to obtain highly performed CMs [10,11,12]. The average distance between the conducting fillers in the network plays a crucial role in the mechanism and level of conductivity of the final composition.

Usually, a high content of conductive fillers is required to achieve reasonable conductivity in cases of random distribution of fillers in a polymer matrix. In many cases, it promotes the high viscosity of the composite mixture and, as a result, the brittleness and enhanced porosity of the composite due to the aggregation of the fillers [13]. The electrical conductivity of such composites with the already formed conductive network is sufficiently lower (5–7 orders of magnitude) compared with a conductivity of the initial fillers, carbon nanotube, carbon black and graphite nanoplatelets. This is because the possible aggregation force of the fillers, including van der Waals force, liquid bridge, electrostatic force, and hydrogen bonding, is not large enough to repel all polymer away in between, and therefore the network by such a self-assembly mechanism cannot be very compacted.

Now, several approaches are used for the formation of controlled conductive fillers’ distribution in the polymer matrix and the development of composites with highly efficient conductive networks [14,15,16]. The creation of polymer composites with a segregated filler network is a promising way to achieve the high electrical conductivity in such composites [17,18] at relatively low nanofiller content. The general idea of the synthesizing of the composites with segregated networks is as follows. In regular polymer composites, the conductive particle coordinate *r* is considered as a continuously changing variable in *V_tot_*, limited only by the physical boundaries of the composites. For the case of the segregated composites, the total composite volume *V_tot_* is divided into two sub-regions: *V^+^*, where particles can appear, and *V^−^*, where particles are forbidden. The local filler concentration in *V^+^* is significantly higher, and this allows the reduction of the total filler concentration in *V_tot_*. 

Experimentally, there are several ways to develop *V^−^*: introduction of non-conductive particles into the composite [19], and ceramic materials, where carbon nanotubes (CNTs) are situated only between grains [20]. However, these methods deal with multi-phase systems, and the production and analysis of the materials become more complex. In the case of polymer-based, there are several approaches to create segregated network, only for a two-phase composite. The first one is a pressing of the mixture of the polymer powder and the conductive filler [21]. Second is using the blends of polymers [22,23,24]. In this case, CNTs are predominantly distributed inside the first polymer, and further, it mixed with the second polymer. The most recent method is using a polymer emulsion to create the segregated network [25,26].

As was shown in [27,28] for concentration range above the percolation threshold in CMs, the positive-negative permittivity transition may be observed with moderate values of negative permittivity (contrary to metals with enormously high negative permittivity due to the ultrahigh electron concentration). The investigations performed in [29,30,31] indicated that the arising of the negative permittivity is the result of the oscillation of free electrons in conductive fillers that form the inductive conductive network. The negative permittivity occurs from the dielectric resonance of the polarization [32] or the plasma oscillation of the delocalized electrons in metallic clusters [33]. For example, as was shown for Fe/coated-Fe/epoxy ternary composites (in the frequency range from 10 MHz to 1 GHz) [28], the capacitive isolated coated-Fe particles acted as a building block to control the dispersion of negative permittivity by LC resonance. Since segregated CMs are characterized by the low percolation threshold, we can expect that the transition of positive/negative permittivity may occur for lower conductive filler content, as compared with random CMs. These s-CMs may be interesting as metamaterials for special EMI applications, where the spatial control of the refractive index variations via the structure of the 3D-conductive network may result in a novel behavior of the electromagnetic response.

Composites with a segregated conductive filler network are characterized by relatively high conductivity, resulting in the efficient attenuation of electromagnetic radiation (EMR), due to both reflection and absorption processes [34,35,36]. In [34], authors developed a segregated composite CNT/polylactic acid (PLA), with high electrical conductivity and enhanced shielding efficiency SE = 35.5 dB at CNT content 1.0 wt.% for the X-band frequency range. In [35] researchers synthesized segregated composites ultrahigh molecular weight polyethylene (UHMWPE)/reduced graphene oxide (r-GO) with high values of electromagnetic shielding efficiency SE = 28.3–32.4 dB in the frequency range 8.2–12.4 GHz at low filler content (0.660% of r-GO). 

In recent years, the fabrication of novel segregated polymer composites with hybrid fillers, e.g., nanocarbon/magnetic filler has been extensively performed. In [37] the prepared segregated hybrid PMMA/rGO/Fe_3_O_4_ nanocomposite exhibited remarkable EMI shielding properties, due to a successful combination of the advantages of the segregated structure of rGO and the magnetic properties of magnetite. For example, the PMMA composite, containing 1.1% grapheme + 0.5% magnetite, exhibited EMI shielding of 29 dB for the sample thickness of 2.9 mm in X-band. 

This study presents the results of the investigation of dielectric and shielding properties of segregated polyethylene-based composites filled with multi-walled carbon nanotubes. This includes the measurements of complex impedance in the frequency range 1 kHz–2 MHz and investigation of the reflection and transmission spectra in the frequency range 36–54 GHz. The main aim is to determine the influence of the segregated structure of CNT in polyethylene composites on the electrical and electromagnetic shielding properties.

## 2. Experimental

### 2.1. Materials and CMs Fabrication

The details of segregated composites’ fabrication could be found elsewhere [38,39]. In brief, polyethylene composites with segregated CNT network were prepared by the hot compacting method described in [40]. The following commercially available materials were used:

(i) GHR 8110 ultra-high molecular weight polyethylene UHMWPE Hostalen GUR (PE) pellets by Hoechst AG (Schkopau, Germany) as a matrix. The parameters of UHMWPE are presented in [38,39].
(ii) Cheap Tubes Inc. (Grafton, VT, USA), carbon nanotubes (purity > 90.0%) as a filler, being 10–30 µm by length and 10–30 nm in average outer diameter.

UHMWPE and CNT powders were mechanically mixed by triturating up to the moment when CNTs form a shell layer on the surface of polyethylene pellet. Then, the core-shell particles (i.e., UHMWPE pellets covered with CNT network) were hot-pressed at 160 °C for 5 min at 50 MPa, and then cooled down to the room temperature. The diameter of pressed disk-shaped specimens was 30 mm, while the thickness was 1 ÷ 2 mm.

The segregated composites CNT/PE with filler content of 0.05%; 0.1%; 0.3%; 0.5%; 1.0%; 1.5%; 3.0%; 4.5%; 6%; 10%; 12% were prepared for electrical and electromagnetic shielding measurements.

Microscopic studies of composite samples were performed by using an optical microscope (“Mikmed-1” with ETREK PCM-510 attachment).

### 2.2. Test Methods

The complex dielectric permittivity of prepared segregated polyethylene (PE)-CNT composites in the low-frequency range (1 kHz–2 MHz) was derived from impedance spectra measured for the specimens with diameter 15 mm and thickness 1 mm, using a Z-2000 impedance meter. The study of electromagnetic shielding parameters and microwave permittivity of CNTs/PE segregated CMs within the 40–60 GHz frequency range was performed using Vector panorama Agilent Technologies N5227A-200. The plate-like specimens with lateral size 4.65 × 2.37 mm^2^ and a thickness of 1 mm provide the full coverage of a cross-section of a rectangular waveguide.

The complex relative permittivity was derived from measured S-parameters of material using 85071 Agilent technology software, namely, Transmission Epsilon Polynomial Fit Model.

## 3. Results and Discussion

### 3.1. DC Electrical Measurements

**Figure 1:**
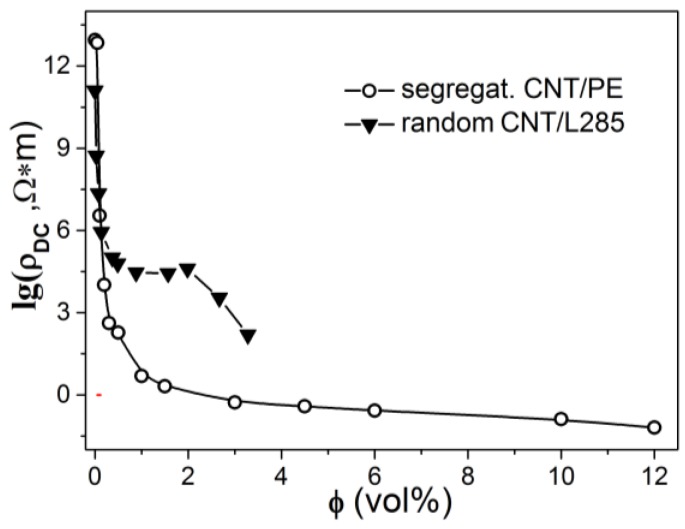
DC resistivity $\rho_{DC}$, versus filler content for composites with segregated carbon nanotubes (CNT)/polyethylene (PE) composites with a segregated filler distribution, and for CNT/epoxy composites with a random filler distribution for comparison.

Figure 1 shows the DC resistance, $\rho_{DC}$, of segregated systems CNT/PE versus CNT content.

One can see that studied structures demonstrate percolation behavior, which can be depicted [41] as ρ=1/σ, where

(1) $$\sigma =\sigma_{f}{\left(\varphi -\varphi_{C}\right)}^{t}\;\mathrm{at}\;\varphi >\varphi_{C}.$$

Here $\varphi_{c}$ is the percolation threshold, *t* is the critical index, whereas $\sigma_{f}$ is the electrical conductivities of CNTs.

The resistivity of CNT/epoxy composites with random CNT filler distribution is also presented for comparison. The detailed description of CNT/epoxy composites fabrication and their electrical properties are presented in our previous paper [42]. The low critical concentration ($\varphi_{c}\;$~0.09%) and high electrical conductivity were observed for the CNT/PE composites, while for the random epoxy CMs the percolation threshold $\varphi_{c}$ is slightly higher (0.13%) and electrical conductivity is much lower. The observed decrease of percolation threshold for segregated CNT distribution compared to a random one agrees with the results for CNT/polyethylene composites presented in the literature. For example, in Ref. [43] three CNT/polyethylene (PE) composites with different conductive networks, i.e., segregated structure (s-CNT/PE), partially segregated structure (p-CNT/PE) and randomly distributed structure (r-CNT/PE) were fabricated. It was shown that s-CNT/PE composite exhibited superior electrical conductivity and percolation threshold $\varphi_{c}$ = 0.013%, which is much lower compared to p-CNT/PE ($\varphi_{c}$ = 0.025%) and r-CNT/PE ($\varphi_{c}$ = 0.310%) composites. In Ref. [44], the segregated composites CNT/PE with a low percolation threshold ($\varphi_{c}$ = 0.054%) and excellent electromagnetic shielding properties have been reported. In our case, the percolation threshold is slightly higher compared to reported ones [43,44], however, it is much lower compared to random CMs [43,44], indicating high efficiency of segregated structure in constructing conductive CNT networks.

**Figure 2:**
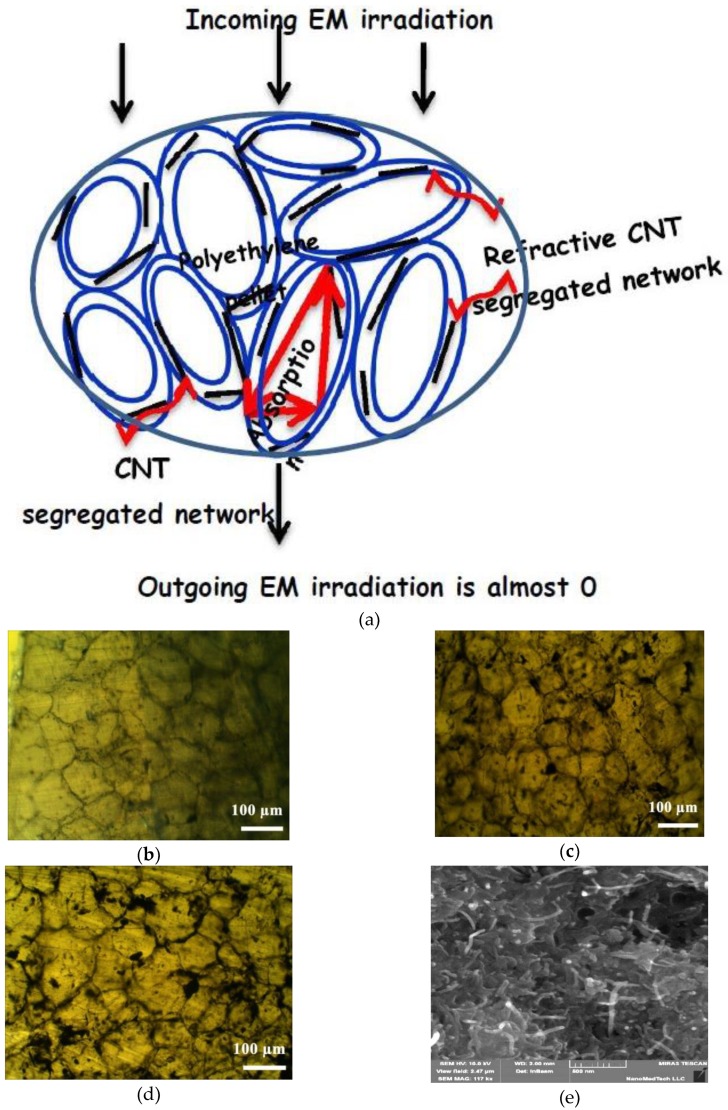
The scheme of segregated structure of composite CNT/PE (**a**), optical images of CNT conductive network formation with increasing filler content in segregated CNT/PE composite materials (CMs): (**b**) 0.05%, below $\varphi_{c}$ (**c**) 0.1%, at $\varphi_{c}$; (**d**) 0.2%, above $\varphi_{c}$ and SEM-image of random 1% CNT/epoxy composite (**e**)

The scheme of the segregated structure of composite CNT/PE interacting with electromagnetic field, optical images of composites CNT/PE filled with various contents of carbon nanotubes and SEM-image of random 1% CNT/polymer composite are shown in Figure 2.

As one can see (Figure 2b–d), the CNT particles are selectively distributed at the interface between polyethylene globules and, as a consequence, the local concentration of CNT particles is much higher compared to CNT content in the whole volume of the composite. Such selective distribution of conductive CNT particles is a result of the large difference in the size and morphology of the composite constituents, as well as the method of fabrication—hot compressing of the mixture of large 3D spherical PE-globules (with size of 90–125 μm) covered with small 1D CNT particles (with diameter 10–30 nm, length 10–30 μm) at the temperature close to PE melting temperature. As it is seen from Figure 2c,d, at CNT content φ is equal to critical concentration $\varphi_{c}$ or higher, the filler pattern becomes noticeable and provides the conductivity in the composite. It is obvious that with increasing CNT content in s-CMs, the segregated CNT-network becomes denser and that improves the contacts between CNT particles and decreases the contact electric resistances in a conductive network. As a result, the segregated CNT-PE composites exhibit the enhanced electrical conductivity compared to composites with random CNTs distribution.

### 3.2. Dielectric Properties

**Figure 3:**
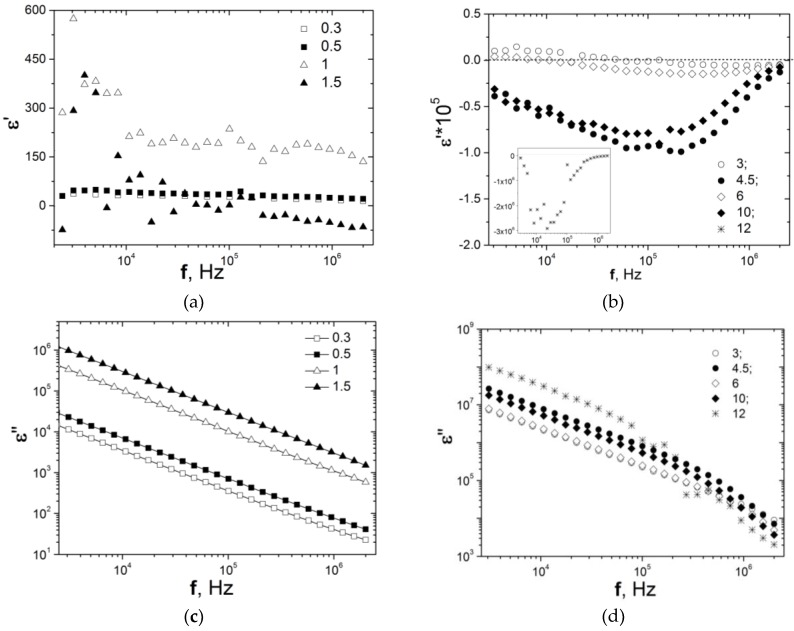
Real (**a,b**) and imaginary (**c,d**) parts of dielectric permittivity of PE-based composites filled with carbon nanotubes versus frequency.

Figure 3 presents the complex permittivity $\varepsilon_{r}^{*}=\varepsilon_{r}^{\prime }-i\cdot \varepsilon_{r}^{\prime \prime }$ of segregated CMs CNT/PE versus frequency. As is seen in Figure 3a, the increase of CNT content up to 1% leads to increase of the real part of permittivity, while for CMs with 1.5% we observed a slight decrease of the dielectric permittivity. The further increase of CNT content leads to the change of sign of permittivity and for samples with CNT content higher than 4.5%, the negative permittivity was observed over the all frequency range 10 Hz–2 MHz, indicating the metal-like electrical conductivity [45,46]. In this case, at CNT content much higher than the percolation threshold, the electrons become delocalized due to formation of the continuous highly conductive network. 

The negative $\varepsilon_{r}^{\prime }$ behavior is attributed to the low frequency plasmon of free electrons in CNT networks [24,45]. In that case, the frequency dispersion of $\varepsilon_{r}^{\prime }$ follows the Drude law [46,47]:

(2) $$\varepsilon_{r}^{\prime }=1-\frac{\omega_{p}^{2}}{\omega^{2}+\omega_{r}^{2}}.$$

(3) $$\omega_{p}=\sqrt{\frac{n_{eff}e^{2}}{m_{eff}\varepsilon_{0}}}.$$

where ω=2πf is the angular frequency of the applied electromagnetic field, $\omega_{p}$ ($\omega_{p}=2\pi f_{p}$) is the angular plasma frequency, $\omega_{r}$ is the damping parameter, $\varepsilon_{0}$ is the permittivity of vacuum (8.85 × 10^−^^12^ F/m ), $n_{eff}$ is the effective concentration of conduction electrons, $m_{eff}\;$is the effective mass of the electron, and e is electron charge (1.6 × 10^−19^ C). As follows from Equation (2), when ω is larger than $\omega_{p}$, the composites behave as an ordinary dielectric medium. However, when ω is smaller than $\omega_{p}$, the real part of permittivity is negative.

The effective electron concentration $n_{eff}$ is determined by the content of CNT in CMs and changes the plasmons frequency and permittivity value according to Equations (2) and (3). As is seen in Figure 3b, the transition from negative to positive is not observed in the studied frequency range (10 Hz–2 MHz) and probably will occur in the higher frequency range for the CMs with a CNT content of 3–12%.

The imaginary part of permittivity ($\varepsilon_{r}^{\prime \prime }$) is a signal of dielectric loss, originated from the conduction process, surface polarization, and dipole movement. Thus, $\varepsilon_{r}^{\prime \prime }$ depends on the frequency and concentration of conductive fillers and reads [30]:

(4) $$\varepsilon_{r}^{\prime \prime }=\varepsilon_{rC}^{\prime \prime }+\varepsilon_{rD}^{\prime \prime }+\varepsilon_{rP}^{\prime \prime }$$

where $\varepsilon_{rC}^{\prime \prime }$ is conduction losses, $\varepsilon_{rD}^{\prime \prime }$ is dipolar losses and $\varepsilon_{rP}^{\prime \prime }$ is interfacial polarization related losses. 

The conduction losses of composites occur from the electric leakage among electroconductive fillers and are described by the following equation [48]:

(5) $$\varepsilon_{rC}^{\prime \prime }=\frac{\sigma_{DC}}{2\pi f\varepsilon_{0}}$$

where $\sigma_{DC}$ is direct current electrical conductivity, constant for a given material.

The polarization currents and the movement of dipoles on continuously changing the electric fields determine the dipolar loss [49].

The relaxation losses mainly arise from the interfacial polarization processes. The charge unbalance, which arises at the CNT/PE interfaces, generates the interfacial polarization. Generally, the interfacial polarization phenomenon occurs at low frequency, which is not more than 1 MHz [50]. Once the frequency of the external electric field reaches a high enough level, the charges do not have time to accumulate at the interface, leading to the disappearance of interfacial polarization.

For all the investigated CNT/PE segregated composites, the $\varepsilon_{r}^{\prime \prime }$ is sufficiently larger (Figure 3c,d), $\varepsilon_{r}^{\prime \prime }$ = 10^4^ for 0.3% CNT/PE and increases up to 10^8^ for 12% CNT/PE and is ascribed to the high conduction loss, while the relaxation losses and interfacial polarization related losses are much smaller [51]. The high value of dielectric losses is the inevitable consequence of the formation of the branched and dense CNT conductive network in the composite. The decrease of the total dielectric losses with frequency is explained by the dominating role of the term $\varepsilon_{rC}^{\prime \prime }$ related to conduction loss (see Equation (5)). Additionally, as is seen in Figure 3c, the graphs of $\varepsilon_{r}^{\prime \prime }$ versus *f* will show a linear decrease relation in a logarithmic scale ($\varepsilon_{r}^{\prime \prime }$ ~ *f*^−1^). The deviation of the curves of $\varepsilon_{r}^{\prime \prime }$ from the linear relationship at the high-frequency regime for the composites at CNT content higher than 4.5% (see Figure 3d) may be explained by the enhancement of the interfacial polarization loss compared to the conduction loss [30,51].

**Figure 4:**
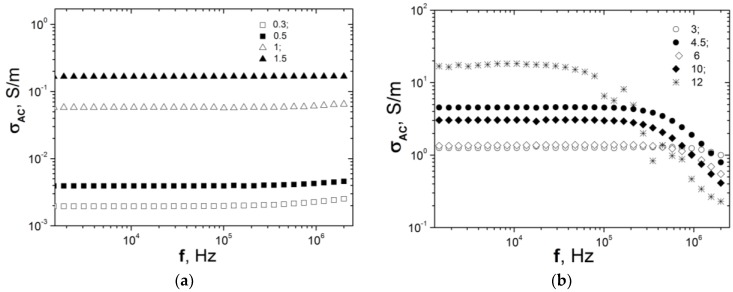
AC conductivity of PE-based composites filled with carbon nanotubes versus frequency. (**a**)—CNT content is 0.3–1.5 vol%; (**b**)—CNT content is 3–12 vol%.

The AC conductivity property of PE/CNT segregated composites with different CNT content is shown in Figure 4. The direct current electrical conductivity ($\sigma_{DC}$) (see Figure 1) shows an increase with CNT content, which indicates that $\sigma_{DC}$ is determined by the density of CNT segregated network and, as a sequence, by the number of conductive pathways and the quality of the electrical contacts between CNT particles.

The rising of carbon nanotubes content leads to the increase of $\sigma_{ac}$, due to the formation of more dense CNT networks for facilitating free carrier transfer, data are presented in Figure 4.

As one can see from Figure 4, for the composites with CNT content lower than 1.5%, the frequency independent conductivity in the low-frequency range is observed and only at the high frequencies $\sigma_{ac}$ is slightly increased. The measured $\sigma_{ac}$ is the sum of two terms, frequently independent DC conductivity and depending on frequency AC conductivity, and is described by an empirical law [52]:

(6) $$\sigma \left(f\right)=\sigma_{DC}+A\cdot \omega^{u}$$

The other composites with CNT content higher than 1.5% (see Figure 4b) show a decrease of $\sigma_{ac}$ conductivity, with increasing frequency beginning from the corresponding critical frequency. Such metal-like conductive behavior (a typical Drude-type response of conductors) is related to the manifestation of the skin effect [52]. In this case, the diffusive electron transport is dominating in a highly conductive dense CNT network formed in segregated CNT/PE CMs.

### 3.3. Microwave Properties of CNT/PE Segregated Composites

**Figure 5:**
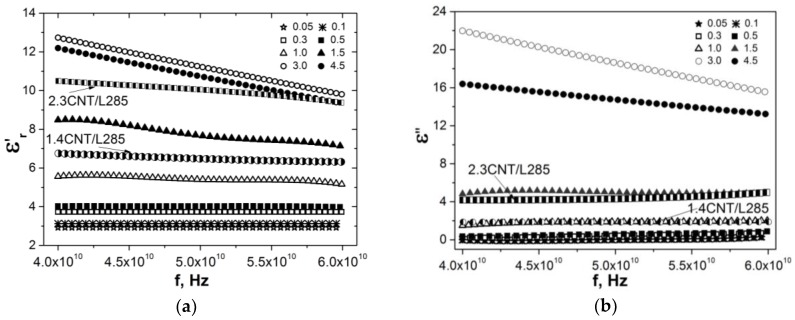
The real $\varepsilon_{r}^{\prime }$ (**a**) and imaginary$\;\varepsilon_{r}^{\prime \prime }$ (**b**) parts of dielectric permittivity for CNT-based segregated and random composites in the frequency range 40–60 GHz.

Microwave properties of segregated CNT/PE composites were studied in the frequency range 40–60 GHz. Using measured S-parameters, the values of complex permittivity were derived and data on $\varepsilon_{r}^{\prime }$ and $\varepsilon_{r}^{\prime \prime }$ are presented in Figure 5.

**Figure 6:**
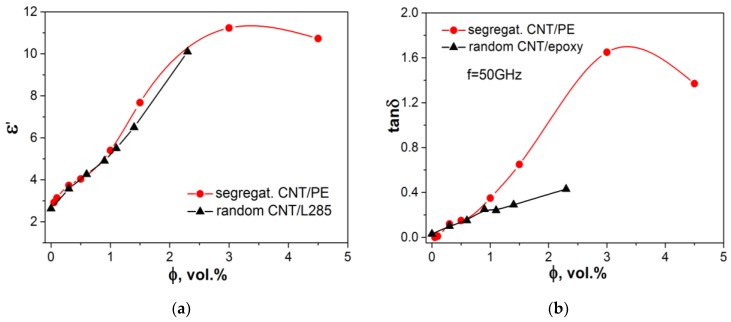
Permittivity $\varepsilon_{r}^{\prime }$ (**a**) and dielectric loss tangent tanδ (**b**) for segregated CNT/PE CMs and random CNT/L285 CMs.

The microwave permittivity for segregated CNT/PE composites is sufficiently lower compared to low-frequency permittivity (see Figure 3) and increases with the increase of CNT content in composite. For comparison, the data on permittivity for previously studied [53] random epoxy composites filled with 1.4% and 2.3% of CNT are presented in Figure 5. Figure 6 displays data on real permittivity and tangent dielectric loss versus CNT content at a frequency of 50 GHz for segregated and random CNT-based composites.

It was found that real permittivity for segregated CMs is slightly higher compared to random CNT-epoxy composite, while the imaginary part of permittivity for s-CMs is sufficiently higher than for random CMs. This difference in imaginary permittivity is related to the high electrical conductivity of segregated CMs, since $\varepsilon_{r}^{\prime \prime }=\sigma /\left(2\pi f\varepsilon_{0}\right)$ and implies the higher EMR absorption loss as well as reflection loss due to a relatively smaller skin-depth for conductive segregated CNT-PE composites compared with random CMs.

Using S-parameters and power balance equation [8,54] EMR reflection *R*, transmission *T* and absorption *A* indices were determined:

(7) $$\begin{array}{ccc} R={\left|S_{11}\right|}^{2} & T={\left|S_{21}\right|}^{2} & A=1-{\left|S_{11}\right|}^{2}-{\left|S_{21}\right|}^{2} \end{array}$$

**Figure 7:**
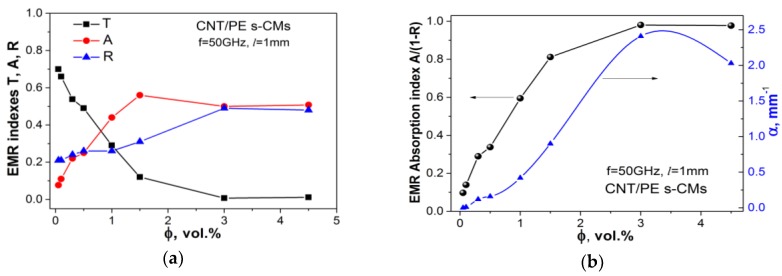
EMR reflection *R*, absorption *A*, transmission *T* (**a**) and EMR absorption indices $A_{eff}$, α (**b**) for CNT/PEs-CMs versus filler content at the fixed frequency 50 GHz. Samples’ thickness is 1 mm.

Figure 7 shows the values of EMR reflection, absorption and transmission indices versus CNT content for CNT/PE s-CMs at a fixed frequency, 50 GHz. The increase of the CNT content in s-CMs causes a sufficient increase of the EMR reflection index and a decrease of the EMR transmission, due to the enhanced electrical conductivity of CMs, and as a sequence, more effective interaction of charge carriers with electromagnetic waves. The most pronounced decrease of the transmission was observed for the composite filled with 3–4.5% CNT. 

Increasing the content of CNT results in absorption index increase, while the ratio of reflection to absorption *R/A* (see Table 1) for segregated PE/CNT CMs composites slightly decreases, that may be explained by an increase of electrical losses in the composite. For the composite filled with 3–4.5% CNT, the ratio *R/A* is increased due to an increase of EMR reflection for these composites.

For the analysis of the EMR absorption efficiency in CMs with segregated fillers distribution, it is convenient to use the reduced absorption index, which may be defined as *A_eff_ = A*/(1 − *R*). Table 1 shows changes in the effective absorption index *A_eff_* versus CNT content for segregated PE/CNT CMs. As can be seen from Figure 7b, the electromagnetic absorption index *A_eff_* increased with CNT content. The increase of CNT concentration up to 4.5% leads to a sharp increase in the effective absorption index *A_eff_*. Such behavior of these indexes can be explained by the high electrical conductivity of s-CM filled with 3–4.5% CNT, which promotes the decrease of skin-depth and the dominant role of EMR reflection of the front boundary air/CM shield. 

The concentration behavior of the absorption losses correlates with the percolation curve for electrical conductivity of investigated s-CM CNT/PE and could be explained by the appearance of large conduction losses in CNT/PE CMs after the percolation threshold. Moreover, in these composites, the segregated CNT network (that was formed as covering of PE globules by CNT particles) acts as numerous reflecting and adsorption conductive interfaces (CNT conductive layers), that effectively interact with electromagnetic radiation. As a result, the excellent microwave shielding and absorbing properties are achieved.

The EMR shielding efficiency $SE_{T}$ (in dB) is defined via the complex index of refraction *n* and propagation constant γ of the electromagnetic waves in the sample (shield) with thickness *l* by the following equation [55]:

(8) $$\begin{array}{l} SE_{T}=-20\log\left|e^{\gamma \cdot l}\right|-20\log\frac{\left|{\left(1+n\right)}^{2}\right|}{4\left|n\right|}-20\log\left|1-\frac{{\left(1-n\right)}^{2}}{{\left(1+n\right)}^{2}}\cdot e^{-2\gamma \cdot l}\right|\\ =SE_{A}+SE_{R}+SE_{MR}\overset{}{,} \end{array}$$

where$\;n=k_{z}/k_{0}$, $k_{0}=2\pi /\lambda_{0}$ is the wave vector in free space, $\lambda_{0}=C_{0}/f$; $\lambda_{0}$ and *f* are the wavelength and the frequency; *C*_0_ = 3 × 10^8^ m/s; $k_{z}=k_{0}\sqrt{\varepsilon_{r}^{*}\mu_{r}^{*}}$; $\varepsilon_{r}^{*}=\varepsilon_{r}^{\prime }-i\varepsilon_{r}^{\prime \prime }$ and $\mu_{r}^{*}=\mu_{r}^{\prime }-i\mu_{r}^{\prime \prime }$ are the relative complex permittivity and relative complex permeability of the medium, respectively; $\gamma =ik_{z}=\alpha +i\beta$, *β* is the phase constant, and *α* is the absorption index.

**Figure 8:**
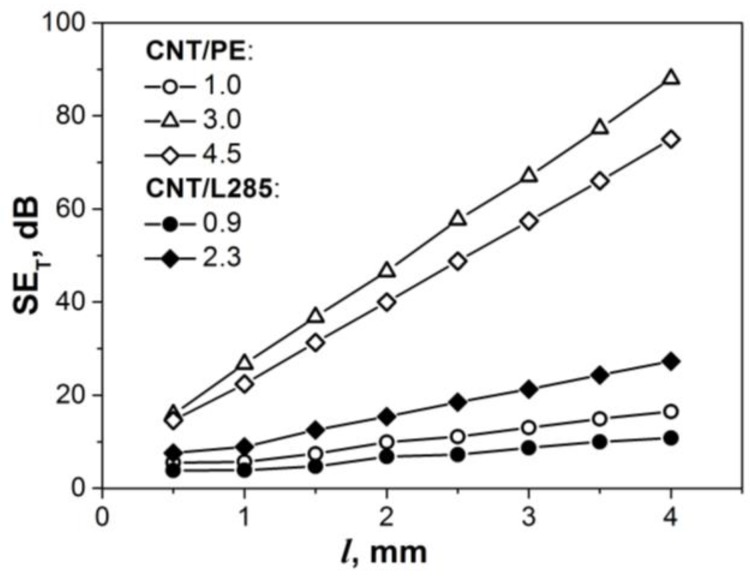
EMR shielding efficiency $SE_{T}$ versus sample thickness *l* for segregated and random CNT-based composites at the fixed frequency 50 GHz.

Using the relation (8) and measured values of complex permittivity the values of EMR shielding efficiency $SE_{T}$ versus thickness *l* for planar, shields were calculated for CNT/PE s-CMs and these data are shown in Figure 8. For comparison, the calculated data for the random CNT/L285 epoxy CMs are also presented. 

It is seen from Figure 8 that electromagnetic wave attenuation for segregated CNT/PE CMs is considerably higher compared to random epoxy composites CNT/L285. This is explained, as was mentioned above, by the increased density and electrical conductivity of CNT layers between PE-globules in segregated composites that provide the higher absorption losses. The shielding efficiency increases linearly with the thickness of composite samples, and for example, s-CMs with CNT content 3–4.5% $SE_{T}$ achieved the values of ~48–57 dB at a sample thickness of 2.5 mm. The estimated absorption index α (data is presented in Figure 7b, right Y-axis) for CNT/PE composites was found to be much larger compared to random CNT/L285 CMs that is a consequence of high dielectric losses (tan*δ* in Figure 6b) and correlates with the effective absorption index *A_eff_*. The archived highly effective EMI shielding ability of segregated CNT/PE composites with valuable content of carbon fillers starting from 3% is the result of multiple reflections on every PE-CNT interface and EM radiation absorbed in each individual CNT-PE granule (see the schematic presentation of the absorption mechanism presented in Figure 2a).

## 4. Conclusions

It was proven that creation of the segregated CNT network in polymer composite leads not only to substantial decrease of the critical percolation concentration compared to CNT/polymer CMs with randomly distributed fillers, but also to the large increase of the absolute values of electrical conductivity of s-CMs, along with almost dispersionless behavior within frequency range from 40 to 60 GHz, covering microwave V-band. The realized outstanding EMI shielding efficiency coursed by absorption mechanism, i.e., A/(1 − R) for 1 mm thick s-CM, is close to 1 at a CNT content of 3–4.5%, and this may be explained by the multiple reflection on every PE-CNT interface, with the subsequent absorption of reflected EM radiation in each individual PE granule encircled by nanotubes that coat. 

The change of the sign of dielectric permittivity $\varepsilon_{r}^{\prime }$ from positive to negative was observed at an increase of CNT content in s-CMs above the percolation threshold and formation of highly electro-conducting CNT networks. The arising of the negative permittivity and AC conductivity behavior versus frequency were related to the low frequency plasmons in conductive CNT networks, and may be described by the Drude model. The observed high values of dielectric losses $\varepsilon_{r}^{\prime \prime }$ in these s-CMs are explained by the domination of high conduction losses above the percolation threshold. 

## Figures and Tables

**Table 1:** Index *A_eff_* and reflection/absorption indexes ratio *R/A* of CNT/PE CMs.

CNT, %	0.05	0.1	0.3	0.5	1.0	1.5	3	4.5
$A_{eff}$, 50 GHz	0.097	0.139	0.289	0.339	0.594	0.812	0.980	0.977
R/A, 50 GHz	2.727	1.909	1.090	1.040	0.591	0.554	0.980	0.945
